# Essentials to Vaccination

**Published:** 1886-02

**Authors:** 


**Essentials of Vaccination.** A compilation of facts relating to vaccine inocculation, and its influence in the prevention of small-pox. By W. A. HARDWAY, M. D., Prof. of Diseases of the Skin in the Post Graduate Faculty of the Missouri Medical College, etc. Pp. 146, 12 mo. St. Louis: J. H. Chambers & Co. 1884.

Dr. Hardaway has collected in this little volume all the more important facts relating to vaccination. Beginning with a history of the subject, the author discusses variola in animals, the nature of vaccinia, the normal and abnormal courses and sequelæ of vaccination, the operation, methods of obtaining and storing virus, and concludes with an examination of the objections to vaccination. We can heartily recommend this book to all of our readers who care to investigate this interesting subject.

